# Composition of gut microbiota involved in alleviation of dexamethasone-induced muscle atrophy by whey protein

**DOI:** 10.1038/s41538-023-00235-w

**Published:** 2023-11-01

**Authors:** JinLing Qiu, Yixing Cheng, Yang Deng, Guangxu Ren, Jiaqi Wang

**Affiliations:** 1 Institute of Food and Nutrition Development, Ministry of Agriculture and Rural Affairs of the Reople’s Republic of China, Beijing, China; 2 College of Food Science and Engineering, Qingdao Agricultural University, Qingdao, Shandong China

**Keywords:** Diseases, Microbial communities

## Abstract

Skeletal muscle atrophy is a condition associated with increased morbidity and mortality. While the concept of the gut-muscle axis has been proposed, the role of gut microbiota in dexamethasone (DEX)-induced skeletal muscle atrophy remains largely unknown, limiting its clinical applications. In this study, we found that administration of DEX caused a shift in the gut microbiota of mice, characterized by an increased ratio of Firmicutes/Bacteroidota and a reduction in alpha diversity. We also identified 480 new operational taxonomic units (OTUs), while 1168 specific OTUs were lost. Our Spearman correlation analysis revealed 28 key taxonomic genera of bacteria that were positively or negatively associated with skeletal muscle strength and weight (r: −0.881 to 0.845, *p* < 0.05). Moreover, supplementation with whey protein reshaped the gut microbiota structure in DEX-treated mice, making it more similar to that of the control group. Importantly, we further utilized a stepwise regression model to identify two enterotypes capable of predicting skeletal muscle function and weight. Notably, *Ileibacterium* and *Lachnospiraceae_UCG-001* played significant roles in predicting both skeletal muscle function and weight. Our findings suggest that DEX causes shifts in the gut microbiota, which can be reversed by whey protein intervention. The enterotypes identified by our stepwise regression models predict muscle function and weight, underscoring the potential role of gut microbiota in modulating muscle atrophy and emphasizing the therapeutic opportunities of microbiota-altering interventions.

## Introduction

The skeletal muscle, as the largest organ and primary protein depot in the body, plays a pivotal role not only in controlling movement but also in various physiological processes including respiration, feeding, energy homeostasis, regulation of nutrient metabolism, and maintenance of high-standard quality of life^1^. Consequently, skeletal muscle atrophy is defined as a reduction in muscle weight and function, which can affect overall metabolism, exercise capacity, eating, and respiration^2^. Importantly, many studies have demonstrated that muscle atrophy is an independent risk factor for mortality^3–5^. Skeletal muscle atrophy is commonly caused by several factors, such as glucocorticoid (GC) administration, aging, denervation, and pathological conditions^6–8^. Numerous pathological conditions characterized by muscle atrophy are associated with increase in circulating GC levels^9^. Furthermore, dexamethasone (DEX), a synthetic glucocorticoid, is frequently utilized for the treatment of inflammatory conditions and allergic diseases, as well as an adjuvant therapy in cancer treatment^10^. Long-term administration of Dex has been shown to decrease the rate of muscle protein synthesis, while increasing the rate of muscle protein degradation. As a consequence, this leads to accelerated onset of muscle atrophy^11,12^. Multiple intracellular mediators, including Akt, mTOR, GSK-3β, β-catenin, FOXO, REDD1, and ATF4, have been implicated in the muscle catabolic and antianabolic effects induced by DEX^13^. Although theoretically blocking the IGF-I or myostatin signaling pathway could prevent DEX-induced muscle atrophy^14^, achieving this objective in clinical practice remains challenging. Currently, the principal approach to mitigate skeletal muscle atrophy is dietary intervention, with whey protein being a representative example^15–17^. It is noteworthy that previous studies on the alleviation of skeletal muscle atrophy by whey protein have primarily focused on supplementing nutrients required for muscle synthesis, such as branched-chain amino acids^14,17,18^. However, this mechanism fails to explain DEX-induced muscle atrophy (non-nutrient deficiency), indicating the existence of alternative mechanisms that require further exploration.

The gut microbiota has recently emerged as a crucial modulator of energy, matter, and information in the human body^19^. Our research group and others have demonstrated that the gut microbiota plays a central role in modulating muscle function and weight^15,20^. Nay et al. employed a perturbation technique using antibiotics (Ab+) to manipulate the gut environment in mice, followed by natural reseeding to restore the gut microbiota. Their findings revealed that the Ab+ treatment led to the depletion of the gut microbiota in mice, resulting in decreased endurance running capacity and impaired ex vivo skeletal muscle contractile function^21^. In a recent study, we demonstrated for the first time in the hematopoietic stem cell transplant (HSCT) population that the composition of the gut microbiota is significantly associated with the effectiveness of whey protein intervention for skeletal muscle atrophy^15^. Furthermore, Haichao Zhao et al. recently observed that DEX administration had a profound impact on the diversity of gut microbiota in mice^22^. This raises the question of whether the gut microbiota participates in the amelioration of DEX-induced skeletal muscle atrophy by whey protein. The gut microbiota possesses a strong plasticity, which can be influenced by modifying dietary structures^23^. Thus, gaining an enhanced comprehension of the crosstalk between gut microbiota and muscle would facilitate the subsequent development of efficacious dietary interventions that alleviate skeletal muscle atrophy. To address this, we aim to utilize a DEX-induced skeletal muscle atrophy mouse and a stepwise regression model to investigate the potential significance of the gut microbiota in the efficacy of whey protein for alleviating DEX-induced skeletal muscle atrophy.

## Results

### DEX induces skeletal muscle dysfunction and atrophy in vivo

**Fig. 1:**
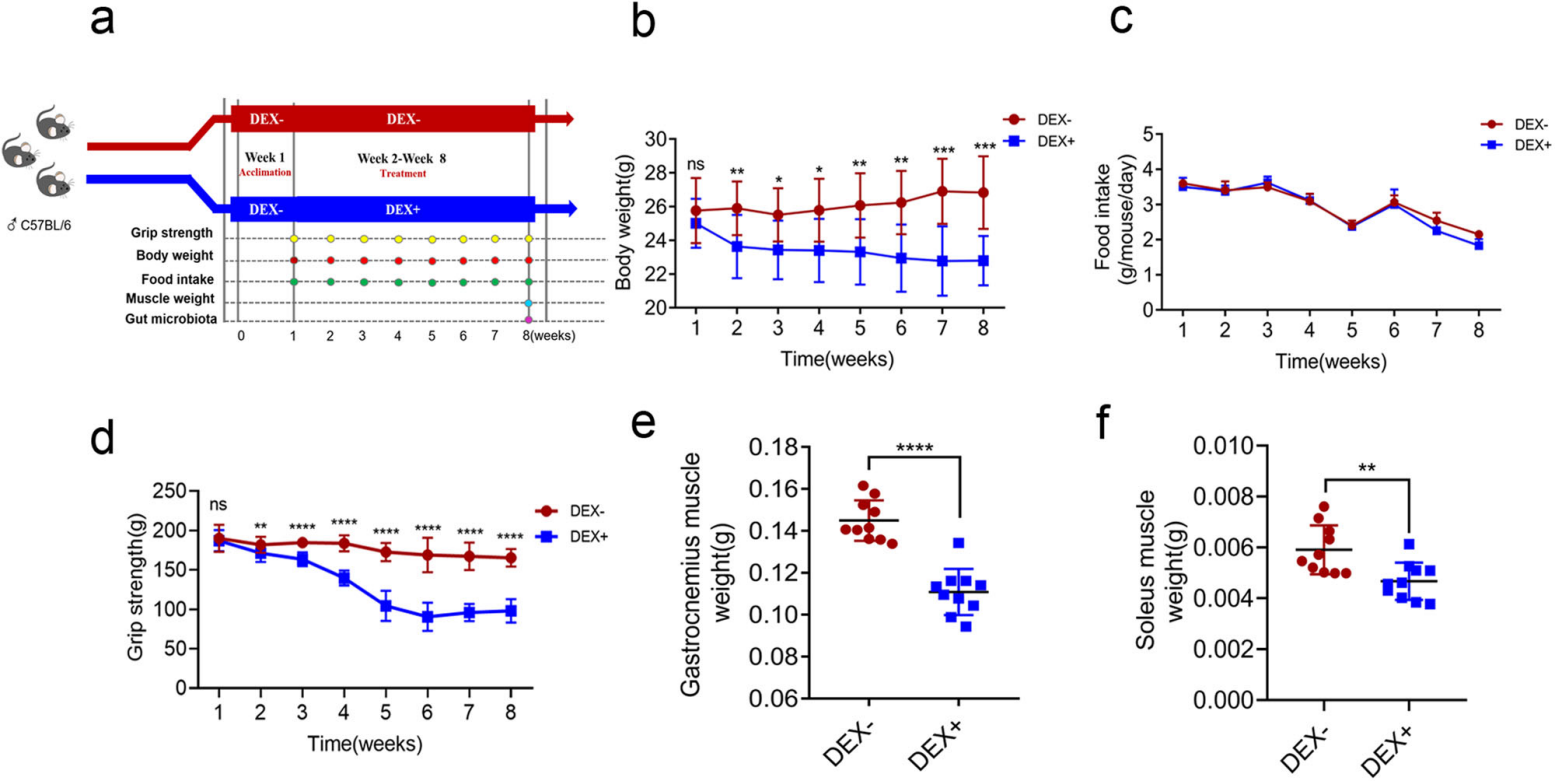
Effect of dexamethasone administration on muscle weight and function. **a** Schematic diagram illustrating the study design. Comparison of body weight **b** and food intake **c** of mice in the DEX- (10 mice) and DEX+ (10 mice) groups from week 1 to week 8. Grip strength dynamics **d** in the DEX- and DEX+ groups from week 1 to week 8. Comparison of gastrocnemius muscle weight **e** and soleus muscle weight **f** between DEX- and DEX+ groups after dexamethasone treatment for 7 weeks. Values are means ± sem. Differences were assessed by independent samples t-test and denoted as follows: ns, no significance; *P < 0.05; **P < 0.01; ***P < 0.001; ****P < 0.0001. (DEX-: without dexamethasone injection; DEX + : dexamethasone injection).

Our analysis revealed a significant decrease in body weight and grip strength in the DEX-treated group at week 2 (*p* < 0.05, Fig. 1b, d), indicating an early loss of skeletal muscle function in response to DEX. However, there were no significant differences in food intake between the DEX- and DEX+ groups at all times (Fig. c). At the end of the session, the weights of gastrocnemius and soleus muscles were significantly decreased by 24.1% and 23.49%, respectively, in the DEX+ group compared to the DEX- group (*p* < 0.05, Fig. 1e, f). These findings corroborate our observations of skeletal muscle function and suggest that DEX leads to the loss of skeletal muscle function and weight.

### DEX-induced alterations in gut microbiota

**Fig. 2:**
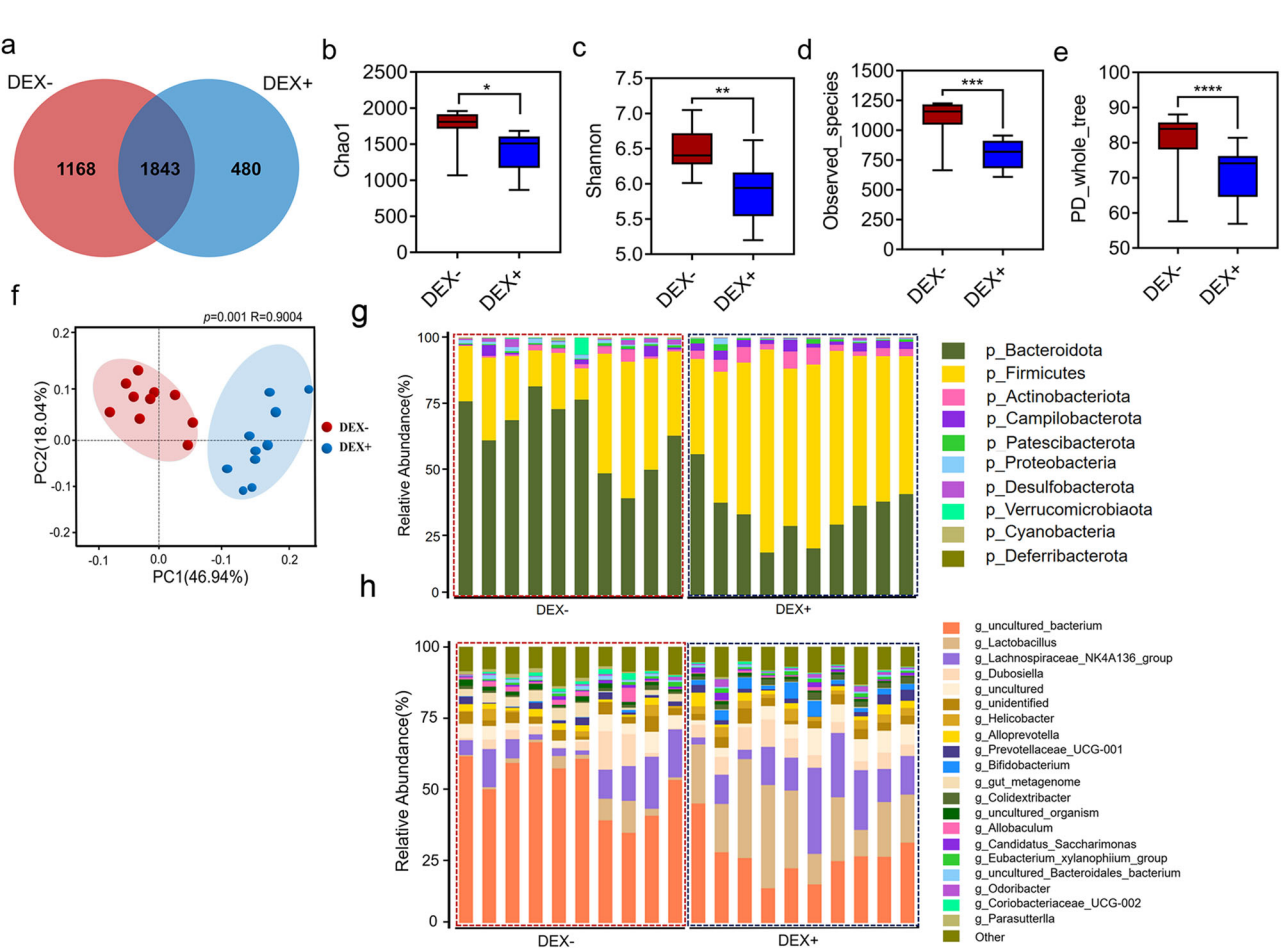
Variation in microbiota due to dexamethasone administration. **a** Venn diagram showing shared operational taxonomic units (OTUs) between DEX- (10 mice) and DEX+ (10 mice) groups at week 8. The alpha diversity of gut microbiota in the two groups of mice was evaluated using Chao1 richness estimator **b**, the Shannon index. **c**, **d** Observed_species, and PD_whole_tree **e**. **f** Principal coordinate analysis (PCoA) based on the Bray-Curtis distance between DEX- and DEX+ groups at week 8. Bar plot depicting the relative abundance of bacteria at phylum **g** and genus **h** between DEX- and DEX+ groups at week 8. Values are means ± s.e.m. Differences were assessed by independent samples *t*-test and denoted as follows: *ns*, no significance; *P < 0.05; **P < 0.01; ***P < 0.001; ****P < 0.0001. The definition of the box plot **b**–**d**: The center line represents the median (second quartile), and the box start and end points represent the first and third quartiles.

Given the high expression of glucocorticoid receptors (GR) in the small intestine and colon^24–26^, we investigated whether intraperitoneal administration of DEX leads to shifts in gut microbiota. We profiled the fecal microbiota of all mice at week 8 by 16 s rRNA gene sequencing. We observed that DEX administration caused the loss of 1168 specific OTUs while leading to the appearance of 480 new OTUs in the intestine of mice (Fig. 2a). Moreover, DEX administration resulted in a significant reduction in alpha-diversity indices of intestinal bacterial communities, mainly reflected in the Chao1, Shannon, observe_species, and PD_whole_tree indices (Fig. 2b–e). Interestingly, the PCoA scatter plot showed a clear clustering of gut bacterial communities between DEX+ and DEX- groups (Fig. 2f). Intraperitoneal injection of DEX was also found to cause definite alterations in the structure of the gut microbiota. Specifically, we compared how DEX administration led to differences in gut microbiota at the phylum and genus levels. Our results showed that DEX administration led to a decrease in the abundance of Bacteroidota and an increase in the abundance of Firmicutes at the phylum level (Fig. 2g). Furthermore, we identified a total of 91 bacterial genera at the genus level, with *uncultured_bacterium*, *Lachnospiraceae_NK4A136_group a*nd *Lactobacillus* being the dominant genera in both groups (Fig. 2h). DEX administration increased the abundance of *Lachnospiraceae_NK4A136_group*, *Lactobacillus* and *Bifidobacterium*, while correspondingly decreasing the abundance of g_unidentified_2 and *Eubacterium_ruminantium_group* (Fig. 2h).

### Association between gut microbiota and skeletal muscle weight and function

**Fig. 3:**
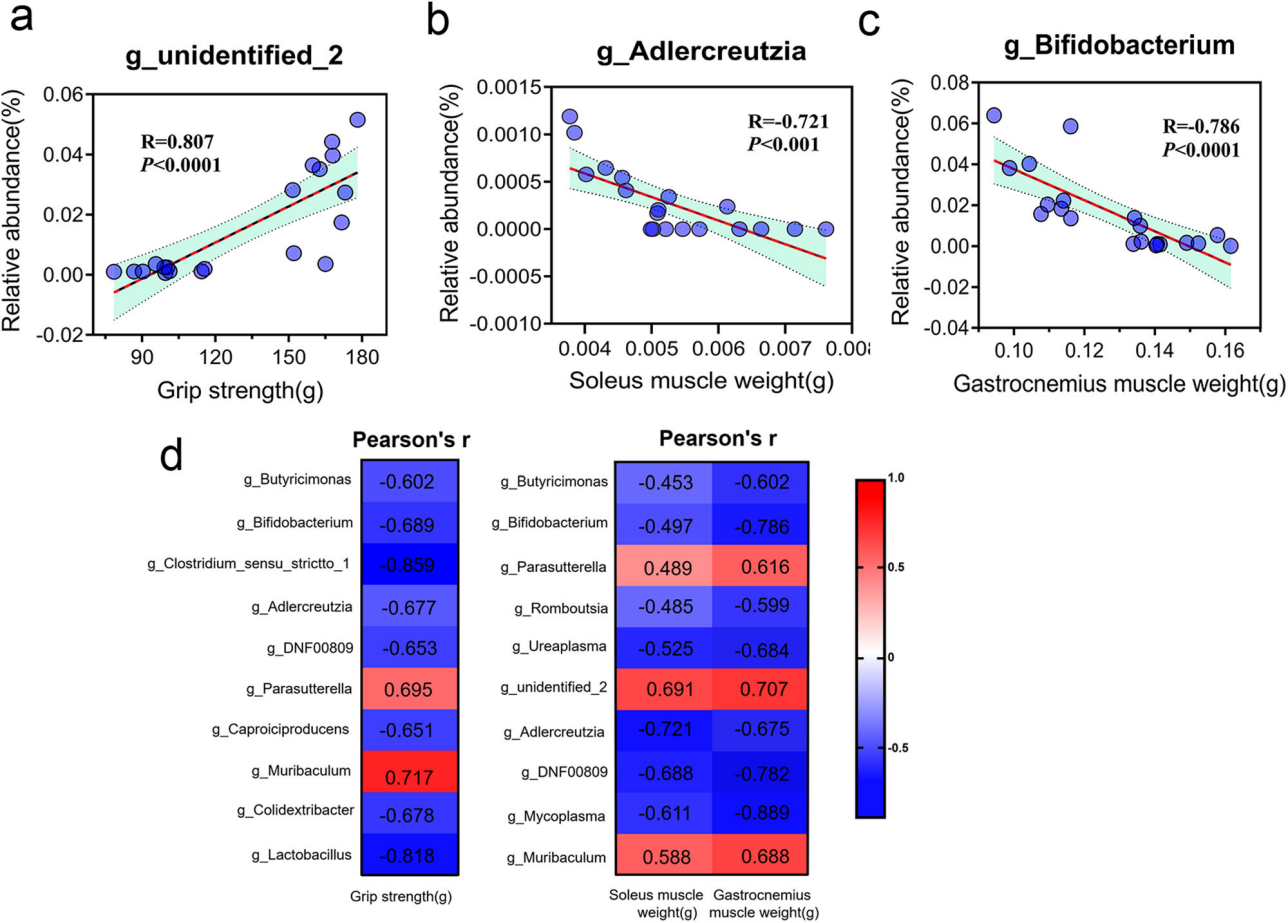
Correlation between gut microbiota and muscle function. Pearson’s correlation analysis between the abundance of the core microbiome and grip strength (**a**, r = 0.807, *p* < 0.0001,95%CI of x-intercept 66.3–108.3), soleus muscle weight (**b**, r = −0.721, *p* < 0.001,95%CI of x-intercept 0.0058–0.0075), and gastrocnemius muscle weight (**c**, r = −0.786, *p* < 0.0001,95%CI of x-intercept 0.14–0.16) in mice (20 mice) at week 8. **d** Pearson’s correlation coefficient (r) for the correlation between core microbiome and grip strength, soleus muscle weight, and gastrocnemius muscle weight. Correlation plot with confidence interval set at 95% (Light green shadowed area).

To investigate the potential involvement of gut microbiota in DEX-induced muscle atrophy, we performed a correlation analysis to examine the association between DEX-responsive gut microbiota and skeletal muscle function and weight. Spearman correlation analysis revealed that 31 key taxonomic genera of bacteria were either positively or negatively correlated with skeletal muscle strength and weight (r: −0.881 −0.881 to 0.845, *p* < 0.05; Supplementary Table 1). Figure 3a–c illustrates a representative result of our correlation analysis. Figure 3a–c illustrates a representative result of the correlation analysis between the abundance of core gut microbiota and skeletal muscle function and weight. Notably, we identified the top 10 bacterial genera at the taxonomic level that were significantly correlated with skeletal muscle grip strength (Fig. 3d), among which *Parasutterella* (r: 0.695) and *Muribaculum* (r: 0.717) displayed a positive correlation with skeletal muscle strength. Furthermore, the abundance of *Parasutterella*, *g_unidentified_2*, and *Muribaculum* showed a significant positive correlation with the weight of both soleus and gastrocnemius (Fig. 3d). Interestingly, both *Parasutterella* and *Muribaculum* exhibited a positive correlation with skeletal muscle function and weight. Conversely, high abundances of *Butyricimonas*, *Adlercreutzia*, and *Bifidobacterium* were found to be negatively correlated with skeletal muscle function and weight. The correlation analysis of other bacterial strains with skeletal muscle function and weight can be found in Supplementary Table 1.

### Whey protein intervention effectively mitigates DEX-induced skeletal muscle dysfunction and weight reduction

**Fig. 4:**
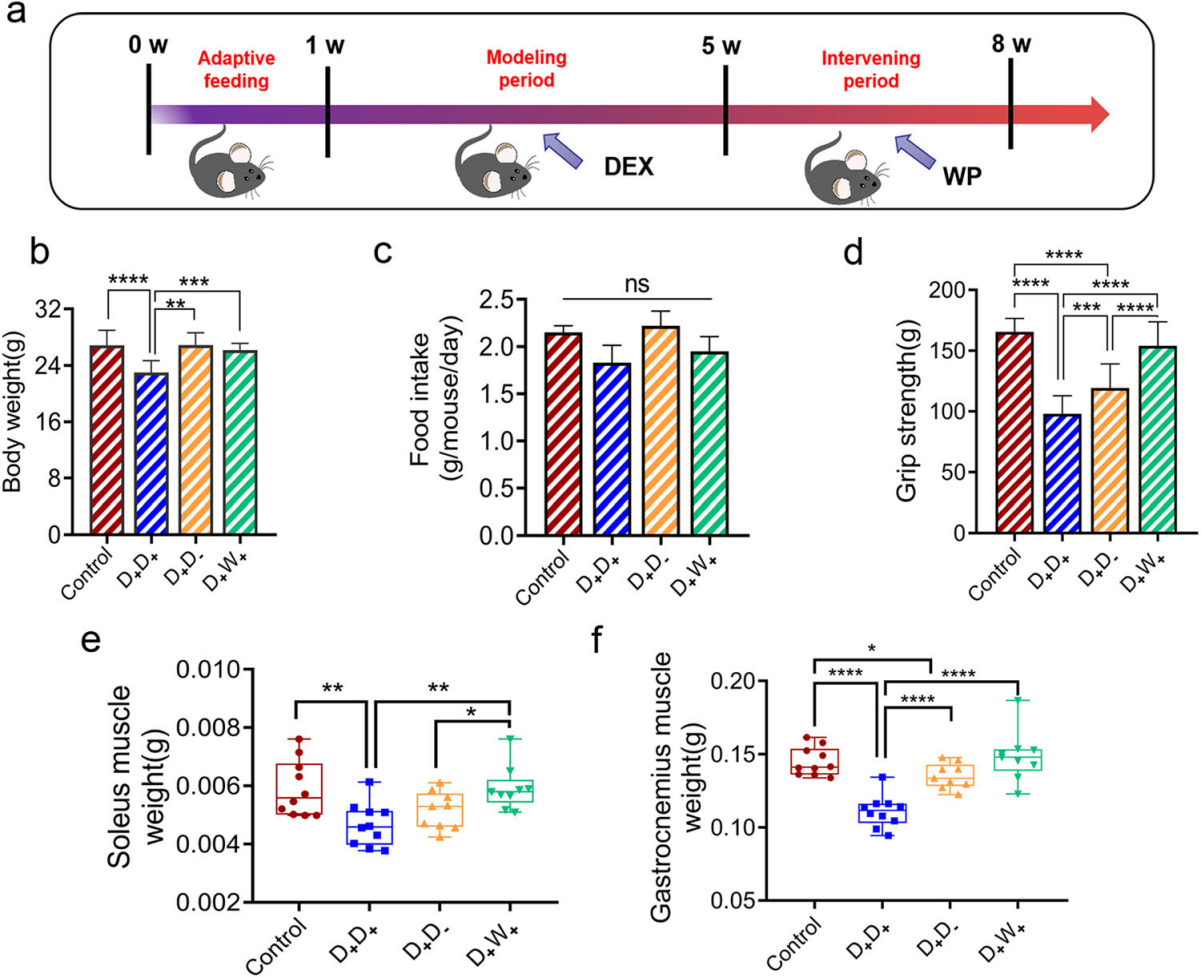
Whey protein intervention improves muscle function in DEX-administered mice. **a** Flow chart of the experimental design for whey protein intervention. Effect of natural recovery (D_+_D_-_,10 mice) and whey protein (D_+_W_+_,10 mice) intervention on body weight (**b**), food intake (**c**), grip strength (**d**), soleus muscle weight (**e**), and gastrocnemius muscle weight (f) of mice after 3 weeks of dexamethasone discontinuation. Values are means ±s.e.m. Differences were assessed by independent sample t-test and denoted as follows: ns, no statistical differences; *P < 0.05; **P < 0.01; ***P < 0.001; ****P < 0.0001.

Given the known ability of whey protein (WP) to promote muscle protein synthesis, we investigated whether WP could alleviate DEX-induced skeletal muscle atrophy (Fig. 4a). To monitor the natural recovery of skeletal muscle function, we discontinued the administration of DEX in mice after three consecutive weeks of treatment (D_+_D_-_). Notably, both the natural recovery group (D_+_D_-_) and the WP group (D_+_W_+_) exhibited a steady increase in body weight upon cessation of DEX. By the eighth week, the body weight in these two groups had returned to levels similar to those observed in the control group (Fig. 4b). Interestingly, despite the weight gain, both the D_+_D_-_ and D_+_W_+_ groups showed significantly lower food intake than the continuous DEX treatment group (D_+_D_+_, Fig. 4c). Ultimately, we observed significantly higher grip strength and muscle weight in the D_+_D_-_ and D_+_W_+_ groups compared to the D_+_D_+_ group (Fig. 4d–f). Moreover, skeletal muscle function and muscle weight in the D_+_W_+_ group were markedly superior to those in the D_+_D_-_ group, with no statistically significant difference in grip strength relative to the control group.

### Impact of interventions on the dynamic changes of gut microbiota

**Fig. 5:**
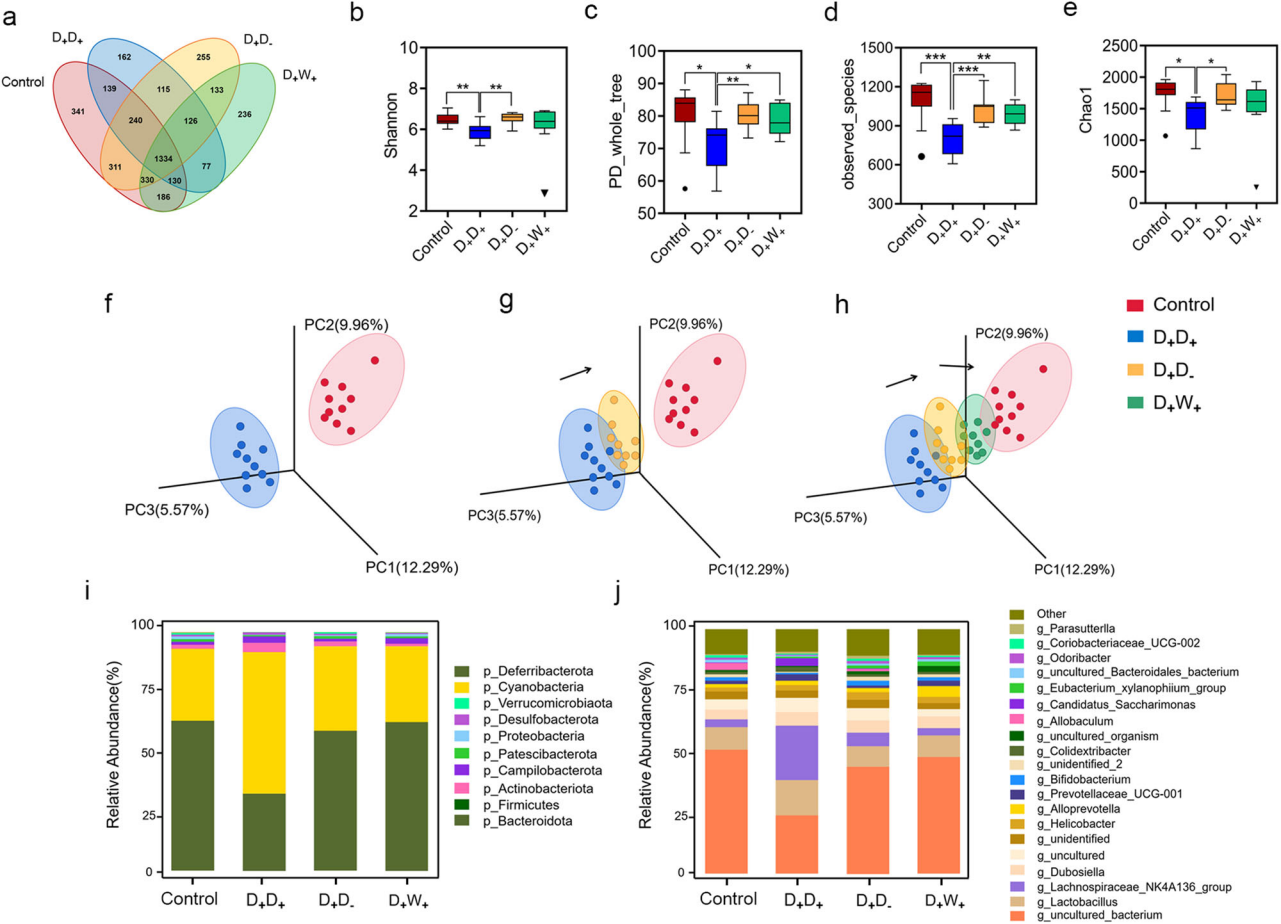
Effect of whey protein intervention on the recovery of intestinal microbial structure in DEX-administered mice. **a** Venn diagram showing shared operational taxonomic units (OTUs) between mice in continuous administration (D_+_D_+_,10 mice), natural recovery (D_+_D_-_,10 mice), and whey protein intervention (D_+_W_+_,10 mice) groups at the 3rd week after dexamethasone discontinuation. The alpha diversity of the microbiota, presented as the Shannon index **b**, PD_whole_tree **c**, observed species **d**, and Chao1 richness estimator **e** between mice in control, D_+_D_+_, D_+_D_-_, and D_+_W_+_ groups at the 3rd week after dexamethasone discontinuation. **f**–**h** Shifts in the gut microbiota of mice between different treatment groups revealed by principal coordinate analysis (PCoA). Relative abundance of gut microbiota in each group at phylum **i** and genus levels **j**. Values are means ± sem. Differences were assessed by independent sample *t*-test and denoted as follows: ns, no statistical differences; *P < 0.05; **P < 0.01; ***P < 0.001; ****P < 0.0001. The definition of the box plot **b**–**e**: The center line represents the median (second quartile), and the box start and end points represent the first and third quartiles.

We next investigated the potential association between the ameliorative effects of WP on DEX-induced skeletal muscle atrophy and the profiles of gut microbiota. We evaluated any alterations in shared bacterial taxa by utilizing Venn analysis when ceasing DEX (D_+_D_-_) and implementing WP (D_+_W_+_) in comparison to the control group or D_+_D_+_ group. Following a three-week cessation of DEX, the D_+_D_-_ group of mice generated 641 new OTU clusters and exhibited the emergence of 641 novel OTU clusters. Upon introducing WP, the D_+_W_+_ group of mice produced 516 new OTUs. These newly emerged OTUs from both groups were also observed in the control group (Fig. 5a). Notably, the D_+_D_-_ and D_+_W_+_ groups of mice shared 133 new OTUs, which were not observed in either the control group or the D_+_D_+_ group. Meanwhile, we observed a significant recovery of gut microbiota diversity in both the D_+_D_-_ and D_+_W_+_ groups, which was not significantly different from the control group (Fig. 5b–e). To monitor the dynamic changes of gut microbiota caused by different treatments, we conducted principal component analysis (PCoA). Interestingly, the gut microbiota structure shifted from the D_+_D_+_ group towards the control group after discontinuation of DEX, with only a few overlapping bacterial clusters between the D_+_D_-_ and D_+_D_-_ mice groups three weeks later (Fig. 5f, g). Notably, the intervention of WP led to a further convergence of the gut microbiota of mice towards the control group, with the gut microbiota of the D_+_W_+_ group being intermediate between the D_+_D_-_ and control groups (Fig. 5h).

Long-term DEX administration reduced the abundance of Bacteroidetes and increased Firmicutes in mouse gut microbiota. However, cessation of DEX or whey protein intervention effectively restored the abundance of Bacteroidetes and Firmicutes to normal levels (Fig. 5i). At the genus level, we found that dexamethasone significantly increased the abundance of *Lachnospiraceae _NK4A136_group*. These data suggest that although the natural recovery group can restore the diversity of gut microbiota, the gut microbiota structure differs significantly from that of normal mice (Fig. 5j).

### Prediction of skeletal muscle function and weight based on gut microbiota composition

**Fig. 6:**
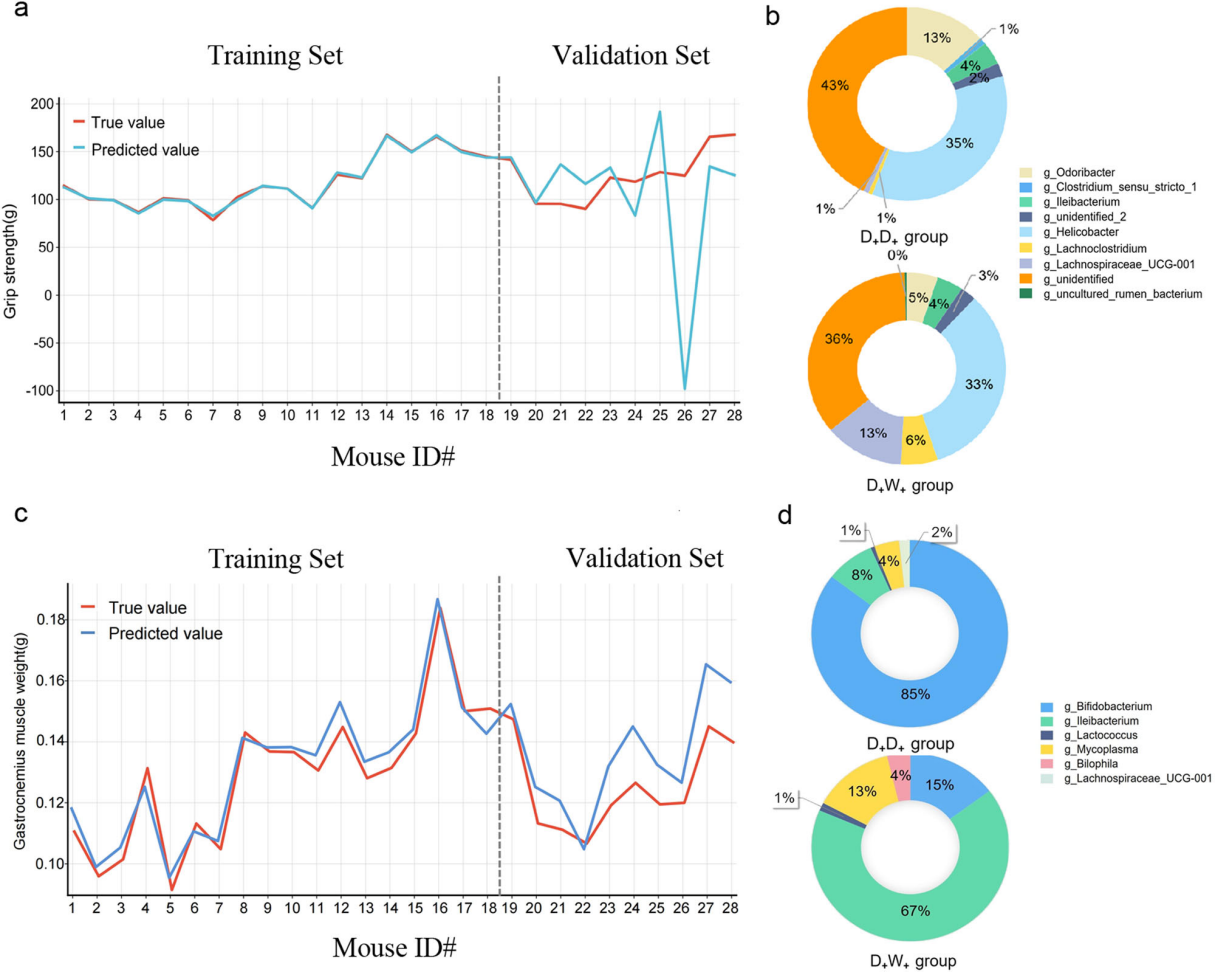
Validation and evaluation of predictive models in muscle weight and function. Training set and validation set of predictive models for grip strength **a** and gastrocnemius muscle weight **c**. Distribution of core bacteria identified in grip strength **b** and the gastrocnemius muscle weight **d** algorithm prediction models between the D_+_D_+_,(10 mice) and D_+_W_+_ (10 mice) groups.

We then sought to determine whether microbiome factors could be incorporated into an algorithm for predicting individualized skeletal muscle function and weight. To this end, we performed a stepwise regression analysis (Supplementary methods). We generated a subset of independent variables that contained key gut bacterial strains and optimized the predictive model for skeletal muscle indicators using a stepwise multiple regression method. A multiple regression model (y = −54897.697X_1_ - 1934.135X_2_ - 9906.16X_3_ - 779.36X_4_ - 2294.91X_5_ + 13319.053X_6_ + 856.594X_7_ - 226.328X_8_ + 272.926X_9_ + 186.771; Model variables (X_n_) and other parameters are provided in Supplemental Table 2) was developed to predict grip strength. When comparing the predicted values to true values, we found that the model could accurately predict changes in mouse grip strength based on their gut microbiota composition pattern, namely enterotype (Fig. 6a). The enterotypes that have the potential to serve as indicators for grip strength prediction include *Clostridium_sensu_stricto_1*, *g_unidentified_2*, *Lachnoclostridium*, *unidentified*, *Ileibacterium*, *uncultured_rumen_bacterium*, *Odoribacter*, *Helicobacter*, and *Lachnospiraceae_UCG-001*. To gain further insight into the contribution of different features in the algorithm’s predictions, we analyzed compositional differences in the gut microbiota of this enterotype present in mice from the D_+_D_+_ and D_+_W_+_ groups (Fig. 6b). To gain further insights into the contribution of different feature bacteria in the algorithm’s predictions, we selected two groups of mice with significant differences in skeletal muscle function and weight, namely the D_+_D_+_ and D_+_W_+_ groups, and analyzed compositional differences in the gut microbiota of this enterotype present in mice. *Helicobacter* was the most abundant genus-level gut bacterium in both D_+_D_+_ and D_+_W_+_ groups, accounting for 35% and 33% of the total bacterial load, respectively. Interestingly, the proportions of *Lachnospiraceae_UCG-001* (D_+_W_+_: 13% v.s. D_+_D_+_:1%) and *Lachnoclostridium* (D_+_W_+_: 6% v.s. D_+_D_+_:1%) were significantly higher in D_+_W_+_ group than in D_+_D_+_ group, while the proportion of Odoribacter (D_+_W_+_: 5% v.s. D_+_D_+_ 13%)was significantly higher in D_+_D_+_ group than in D_+_W_+_ group. We further optimized the stepwise regression model to identify the enterotype that can accurately predict skeletal muscle weight (y = −0.508X_1_ + 52.558X_2_ + 1.782X_3_ + 0.914X_4_ - 7.009X_5_ - 21.799*X_6_ + 0.136；X_n_ and other parameters are provided in Supplemental Table 2). Similarly, we found the enterotype composed of *Bifidobacterium*, *Bilophila*, *Ileibacterium*, *Lachnospiraceae_UCG-001*, *Mycoplasma*, and *Lactococcus* was effective in predicting the weight of gastrocnemius muscle (Fig. 6b). Notably, we identified different predominant bacteria in the two groups, with *Bifidobacterium* being the most abundant in the D_+_D_+_ group (85%) and *Ileibacterium* being the most abundant in the D_+_W_+_ group (67%).

## Discussion

The significance of maintaining muscle health lies in the fact that skeletal muscles serve as primary sites for insulin-mediated glucose uptake, thereby exerting a critical influence on glucose homeostasis and overall metabolic function in the body. However, there are several factors that can lead to skeletal muscle atrophy. Among these, glucocorticoid-induced skeletal muscle atrophy is the most severe and rapid, with no effective relief method. The gut microbiota composition and microbial metabolites have been investigated in various disease contexts due to the bidirectional communication between the gut microbiota and their host. In recent years, mounting evidence has suggested that the gut microbiota, serving as an important metabolic hub, may be involved in the regulation of skeletal muscle^25–27^. In patients undergoing hematopoietic stem cell transplantation, we observed a significant correlation between the composition of gut microbiota and skeletal muscle function. This finding suggests the existence of a regulatory pathway between the gut and muscle^15^.

In the present study, we utilized the DEX mouse model to investigate the effect of DEX on the composition of gut microbiota and its impact on skeletal muscle function and weight. Our results suggest that DEX significantly affects the structure of gut microbiota, leading to a decrease in alpha diversity. Although the gut microbiota of the natural recovery group (D_+_D_-_) and whey protein intervention group (D_+_W_+_) tended to return towards the microbiota structure of the normal control group, the D_+_W_+_ group exhibited the closest resemblance to the microbiota structure of the normal group among the groups. Furthermore, we demonstrated that a stepwise regression algorithm that integrates microbiome features can accurately predict skeletal muscle function and weight.

The mechanism by which DEX promotes muscle atrophy is still not well understood. Currently, the focus of research on the induction of skeletal muscle atrophy by DEX is primarily on exploring the imbalance between skeletal muscle protein synthesis and degradation at the level of signal transduction pathways^26^. To date, no targeted drug has been developed to counteract skeletal muscle atrophy and alleviate the adverse effects of DEX on skeletal muscle. Interestingly, gut microbiota has recently been proposed as an environmental factor involved in various physiological functions, including energy sparing from the diet and regulation of host skeletal muscle metabolism^27^. Our data revealed that the administration of DEX induced a shift in the gut microbiota of mice, accompanied by an increase in the ratio of Firmicutes/Bacteroidetes and a decrease in the alpha-diversity of the gut microbial community. Notably, most Bacteroidetes in the gut harvest energy from indigestible polysaccharides and produce short-chain fatty acids (SCFAs), which regulate host energy metabolism^28^. Administration of a mixture of these SCFAs to Germ-Free mice was shown to partly reverse skeletal muscle impairment caused by gut microbiota deficiency, notably through the improvement of muscle strength^29^. Considering the high plasticity of gut microbiota and its regulation by environmental factors such as diet^30^, exploring the regulatory mechanisms of DEX-induced skeletal muscle atrophy from the perspective of gut bacteria is a crucial step toward the development of functional foods aimed at mitigating DEX-induced skeletal muscle atrophy in the future.

Nutritional intervention is considered a relatively effective way to improve muscle atrophy^31^. Direct and indirect evidence suggests that whey protein (PRO) may be an especially suitable pre- or post-exercise ingestion to be used in conjunction with resistance exercise to stimulate muscle hypertrophy^32^. Our research group and others have found that whey protein has a well-balanced amino acid composition, with a high content of branched-chain amino acids (BCAAs)^33^. Given that BCAAs, including leucine, isoleucine, and valine, can enhance muscle anabolic signaling, previous studies on the mechanism of whey protein improving muscle function have mainly focused on providing essential substrates for skeletal muscle synthesis. However, for non-nutritional deficiency types of muscular atrophy, such as DEX-induced atrophy, it is difficult to explain the improvement mechanism solely through the theory of supplementing skeletal muscle synthesis materials. The main purpose of using whey protein intervention in this study is to observe whether whey protein can restore the gut microbiota dysbiosis induced by DEX. The PCA data indicate that whey protein intervention can improve the gut microbiota structure, which has been altered by DEX, towards that of normal mice. Moreover, the structural changes are significantly greater than those observed in the group of mice undergoing natural recovery. Importantly, whey protein intervention effectively restored the abundance of Bacteroidetes and Firmicutes to normal levels. The interindividual variability of the gut microbiome is substantial, especially when considering the relative changes in both dominant and rare taxa. By leveraging deep sequencing technologies, this variability has been linked to a wide range of diseases^34^. Understanding the characteristics of microbial diversity in healthy individuals, investigating modifications of the enterotype during skeletal muscle atrophy, and exploring the potential utilization of microbial features for predicting certain conditions represent significant challenges in the current scientific field.

Enterotype analysis has the potential advantage of identifying possible correlations between enterotypes and various diseases. We expect a stepwise regression model to be effective in determining the enterotypes related to skeletal muscle function. In the present study, we utilized this model to determine two enterotypes capable of predicting skeletal muscle function and weight. Interestingly, the microbial composition strains that make up these two enterotypes are not entirely identical. Notably, both Ileibacterium and Lachnospiraceae_UCG-001 are present in both enterotypes. Currently, little is known about the function of Ileibacterium. However, some preliminary studies suggest that Ileibacterium may be involved in metabolism, as its expression level has been shown to be negatively correlated with hydrophilic bile acids^35^. Interestingly, *Bifidobacterium*, *Ileibacterium*, and *Lachnospiraceae_UCG-001* are capable of producing short-chain fatty acids (SCFAs)^36–38^. These volatile fatty acids contain fewer than six carbons and have well-established effects on whole-body energy homeostasis. SCFAs are absorbed from the gut lumen and modulate host metabolic responses at different organ sites. Evidence suggests that these organ sites include skeletal muscle, the largest organ in humans, which plays a pivotal role in whole-body energy metabolism. Increasing evidence suggests that SCFAs mediate metabolic cross-talk between the gut microbiota and skeletal muscle^39^.

This study used a small sample size of gut microbiota data combined with a stepwise regression algorithm to successfully predict skeletal muscle function and weight prospectively. Our data have revealed that the gut microbiota associated with skeletal muscle function and weight demonstrates a distinct enterotype, and the core bacterial species may be interconnected in regulatory networks. In order to apply this study to the clinic, we will collect in vivo data for clinical studies in the future.

## Methods

### Experimental animal

Six-week-old male C57BL/6 J Specific Pathogen Free (SPF) mice, weighing between 19–22 g, were purchased from Beijing Vitonglihua Laboratory Animal Science and Technology Co., LTD. (Beijing, China). The mice were kept in a specific pathogen-free (SPF) mouse facility with a 12-h light-dark cycle at a room temperature of 18–23 °C and 45–55% relative humidity for acclimatization. They were given the standard growth maintenance chow feed (GMCF purchased from Beijing Keao Xieli Feed Limited Company, Beijing, China; Product ID: 1016706714625204224; GB14924.3-2010, China National Standard) and water ad libitum. The nutrients of GMCF were described in our previous work^33^. Whey protein (250 g/bottle) was obtained from Yuanye Biotechnology Co., Ltd. The whey protein was dissolved in deionized water to prepare a 0.3 g/mL solution and gavaged to the mice at a dose of 0.5 mL per mouse. Dexamethasone (DEX,100 mg; Solarbio, China) was dissolved in 2 mL of dimethyl sulfoxide (DMSO) solvent.

All experimental procedures involving animals were performed in accordance with protocols approved by the Institutional Animal Care and Usage Committees (IACUC) of the Institute of Food and Nutrition Development, MARA. The approval number for these procedures is YYSLLSC20200047.

### Experimental design

After one week of acclimatization with standard rodent maintenance feed, the C57BL/6J mice were randomly divided into two groups: (1) DEX_-_ group (*n* = 10), in which normal saline (0.9%) was administered intraperitoneally every other day; (2) DEX_+_ group (*n* = 30), in which muscle atrophy model was constructed randomly selected from the pool of mice. The mice in the DEX_+_ group were intraperitoneally injected with DEX (25 mg/kg) every other day for four weeks to induce muscle atrophy. After the completion of muscle atrophy modeling, all DEX-treated mice were divided into three groups and subjected to an additional three weeks of experimentation: (1) D_+_D_+_ group (*n* = 10), in which DEX injection was continued intraperitoneally at the same dose as before; (2) D_+_D_-_ group (*n* = 10), in which DEX injection was discontinued after successful modeling, mainly to observe the natural recovery condition of muscle; (3) D_+_W_+_ group (*n* = 10), in which whey protein intervention (administered by gavage at a dose of 0.3 g/ml) was given to the mice after discontinuation of DEX injections in an attempt to ameliorate the DEX induced muscle atrophy. Ten mice were housed in each group, with five animals in each cage. Grip strength, body weight, food intake, muscle weight, and gut microbiota were measured at designated time points (as indicated in Fig. 1a).

### Parameters related to skeletal muscle function and weight

Grip strength was measured using a grip strength meter (YLS-13A, Jinan Yiyan Technology Development Co., Ltd, Shandong, China). The mice were held by the tail, and their forelimbs were allowed to grasp the wire mesh. Subsequently, the mice were gently pulled backward until they released their grip on the grid. Six valid values were obtained for each group, and the average grip strength was used for statistical analysis. At the end of the intervention period, the skeletal muscles (gastrocnemius and soleus) were dissected from the carcass and trimmed of all fat, fascia, and connective tissue. The wet weight of each muscle was immediately measured and recorded. Body weight and food intake were monitored and recorded weekly at predetermined time points throughout the study. Fresh stool samples were collected from each mouse at the endpoint of the study and immediately stored in sterile tubes containing liquid nitrogen to await sequencing analysis.

### Gut microbiota analysis

The extraction of DNA from cecal contents and 16 S rRNA gene sequencing was performed as described previously^15^. The sequences with high similarity (97%) were classified into one OTU, and the obtained valid Tag sequences were clustered, so as to compare and analyze the OTU of mouse colon contents. The complexity of species diversity(α-diversity) was analyzed by calculating Chao 1, Shannon, PD_whole_tree, and Simpson indices. Changes in community composition were measured by calculating principal component analysis (PCA). In addition, the relative abundance of dominant bacteria at phylum and genus levels was also analyzed.

### The gut microbiota-based predictive model

Multifactor analysis was conducted using stepwise regression analysis (Detailed methods are provided in supplementary methods). This approach involves constructing a model with all potential dependent variables and then gradually eliminating variables to retain the model with the highest coefficient of determination while ensuring the significance of parameters^40^. The criteria for variable entry and removal in the stepwise regression analysis were based on a significant F-value of 0.05. The optimal equation for the model was determined by the highest multiple correlation coefficient (R^2^). The multiple regression model used in this study is represented by the following formula (1):

1 $${\rm{y}}={{\rm{a}}}_{0}+{{\rm{a}}}_{1}{{\rm{x}}}_{1}+{{\rm{a}}}_{2}{{\rm{x}}}_{2}+{{\rm{a}}}_{3}{{\rm{x}}}_{3}+{{\rm{a}}}_{4}{{\rm{x}}}_{4}+\ldots +{{\rm{a}}}_{{\rm{n}}}{{\rm{x}}}_{{\rm{n}}}$$

In this study, the stability of the model was assessed using the coefficient of determination (R^2^), while its quality was evaluated using the root mean square error (RMSE). A higher R^2^ value indicates better predictive ability of the model, while a lower RMSE value indicates more accurate fitting of the model^40^. The formula for calculating RMSE is as follows:

2 $${\rm{RMSE}}=\left[\frac{{\sum }_{{\rm{i}}=1}^{{\rm{n}}}({{\rm{Pi}}-{\rm{Oi}})}^{2}}{{\rm{n}}}\right]$$

To validate the model, the obtained value was compared with the true value using an independent dataset that was not used to construct the regression model. The detailed process can be found in the supporting document.

### Statistical analysis

The data were analyzed using a combination of the software programs *R* and Mothur. Heat maps, principal coordinate analysis plots, and bar plots were created in *R* using the packages vegan and g plots. Two-sided analyses of nonparametric Kruskal-Wallis test, Fisher’s exact rect, and t-test were performed using GraphPad Prism software version 8.0.0 (GraphPad Inc., San Diego, California, USA) and SPSS software version 26 (IBM Corp., Armonk, NY, USA). A prediction model of skeletal muscle atrophy was developed based on gut microbiota using stepwise regression analysis. The criterion for statistical significance was set at *p* < 0.05.

### Reporting summary

Further information on research design is available in the Nature Research Reporting Summary linked to this article.

### Supplementary information

Supplemental Table

Reporting Summary

## Data Availability

The sequence data have been deposited in the NCBI Sequence Read Archive under BioProject (PRJNA932250).
