# Supplementary material for: Composition of gut microbiota involved in alleviation of dexamethasone-induced muscle atrophy by whey protein
Source: NPJ Sci Food. 2023 Nov 1;7:58. doi: 10.1038/s41538-023-00235-w (PMC10618183; doi:10.1038/s41538-023-00235-w)
Supplement: Supplementary file 1 — Supplemental Table [file 41538_2023_235_MOESM1_ESM.doc]

**Supplementary Tables**

**Supplemental Table 1**

**The correlation between genus-level bacteria and the weight of soleus muscle, gastrocnemius muscle, and grip strength**

| **Soleus muscle weight (n=20)** | | | **Gastrocnemius muscle weight (n=20)** | | | **Grip strength (n=20)** | | |
| --- | --- | --- | --- | --- | --- | --- | --- | --- |
| Genus-level bacterial name | Pearson correlation | Sig.([two-tailed test](javascript:;)) | Genus-level bacterial name | Pearson correlation | Sig.([two-tailed test](javascript:;)) | Genus-level bacterial name | Pearson correlation | Sig.([two-tailed test](javascript:;)) |
| Butyricimonas | -0.453 | 0.045 | Clostridium_sensu_stricto_1 | -0.753** | *P*<0.01 | Romboutsia | -0.565** | 0.009 |
| uncultured_Bacteroidales_bacterium | 0.620** | 0.004 | Acetitomaculum | -0.447 | 0.048 | Ureaplasma | -0.559 | 0.01 |
| Harryflintia | 0.507 | 0.022 | Monoglobus | -0.485 | 0.03 | Eubacterium_siraeum_group | -0.547 | 0.013 |
| Prevotellaceae_Ga6A1_group | 0.595** | 0.006 | Rikenella | -0.456 | 0.043 | Monoglobus | -0.481 | 0.032 |
| UCG-005 | -0.506 | 0.023 | Moryella | -0.473 | 0.035 | Bacteroides | 0.526 | 0.017 |
| Caproiciproducens | -0.461 | 0.041 | Escherichia-Shigella | -0.493 | 0.027 | Lactococcus | -0.511 | 0.021 |
| Colidextribacter | -0.465 | 0.039 | uncultured_Bacteroidales_bacterium | 0.544 | 0.013 | ASF356 | 0.469 | 0.037 |
| Lactobacillus | -0.500 | 0.025 | uncultured_organism | 0.452 | 0.045 | gut_metagenome | 0.807** | *P*<0.01 |
| uncultured_bacterium | 0.553 | 0.011 | Desulfovibrio | 0.592** | 0.006 | Rikenella | -0.528 | 0.017 |
| - | - | - | Helicobacter | -0.594** | 0.006 | Escherichia-Shigella | -0.474 | 0.035 |
| - | - | - | Harryflintia | 0.666** | 0.001 | uncultured_Bacteroidales_bacterium | 0.793** | *P*<0.01 |
| - | - | - | Rikenella | -0.446 | 0.048 | uncultured_organism | 0.526 | 0.017 |
| - | - | - | Caproiciproducens | -0.709** | *P*<0.01 | Desulfovibrio | 0.550 | 0.012 |
| - | - | - | Christensenellaceae_R-7_group | 0.478 | 0.033 | Mycoplasma | -0.881** | *P*<0.01 |
| - | - | - | Lactobacillus | -0.719** | *P*<0.01 | Harryflintia | 0.531 | 0.016 |
| - | - | - | uncultured_bacterium | 0.744** | *P*<0.01 | Blautia | -0.500 | 0.025 |
| - | - | - | - | - | - | Prevotellaceae_Ga6A1_group | 0.465 | 0.039 |
| - | - | - | - | - | - | UCG-005 | -0.579** | 0.007 |
| - | - | - | - | - | - | Turicibacter | -0.585** | 0.007 |
| - | - | - | - | - | - | Anaeroplasma | -0.465 | 0.039 |
| - | - | - | - | - | - | Caproiciproducens | -0.651** | 0.002 |
| - | - | - | - | - | - | Muribaculum | 0.717** | *P*<0.01 |
| - | - | - | - | - | - | Colidextribacter | -0.678** | 0.001 |
| - | - | - | - | - | - | Christensenellaceae_R-7_group | 0.476 | 0.034 |
| - | - | - | - | - | - | Family_XIII_AD3011_group | -0.468 | 0.038 |
| - | - | - | - | - | - | Lactobacillus | -0.813** | *P*<0.01 |
| - | - | - | - | - | - | uncultured_bacterium | 0.845** | *P*<0.01 |
| - | - | - | - | - | - | Faecalibaculum | 0.454 | 0.044 |

Note:***P*<0.01, *Sig.(*[*two-tailed test*](javascript:;)*)*

**Supplemental Table 2**

Table of predicted skeletal muscle mass and functional model variables

| **Y** | **Stepwise regression** | **R** | **R^2^** | **Adjusted R^2^** | **Errors in standard estimates** | **DurbinWatson** | **F** | **parameter** | **Unstandardized coefficient** | **t** | **Sig.** | **VIF** |
| --- | --- | --- | --- | --- | --- | --- | --- | --- | --- | --- | --- | --- |
| **Gastrocnemiusmuscle weight** | y=-0.508*g_Bifidobacterium+ 52.558*g__Bilophila+ 1.782*g__Ileibacterium+ 0.914*g__Lachnospiraceae_UCG-001- 7.009*g__Mycoplasm-21.799*g__Lactococcus+0.136 | 0.983 | 0.966 | 0.949 | 0.005179545 | 1.951 | 56.637 | Constant | 0.136 | 56.414 | 0.0001 | - |
|  |  |  |  |  |  |  |  | g__Bifidobacterium | -0.508 | -4.922 | 0.0001 | 3.003 |
|  |  |  |  |  |  |  |  | g__Bilophila | 52.558 | 4.891 | 0.0001 | 1.791 |
|  |  |  |  |  |  |  |  | g__Ileibacterium | 1.782 | 4.730 | 0.0001 | 1.217 |
|  |  |  |  |  |  |  |  | g__Lachnospiraceae_UCG-001 | 0.914 | 4.241 | 0.001 | 1.818 |
|  |  |  |  |  |  |  |  | g__Mycoplasm | -7.009 | -2.435 | 0.031 | 3.038 |
|  |  |  |  |  |  |  |  | g__Lactococcus | -21.799 | -2.32 | 0.039 | 1.383 |
| **Grip strength** | y=-54897.697**g_Clostridium_sensu_stricto_1*-1934.135**g_gut_metagenome*-9906.16**g_Lachnoclostridium*-779.36**g_unidentified*-2294.91**g_Ileibacterium*+13319.053**g_uncultured_rumen_bacterium*+856.594**g_Odoribacter*-226.328**g_Helicobacter*+272.926**g_Lachnospiraceae_UCG-001*+186.771 | 0.998 | 0.996 | 0.992 | 2.382 | 2.537 | 255.229 | Constant | 186.771 | 63.381 | 0.0001 | - |
|  |  |  |  |  |  |  |  | g__Clostridium_sensu_stricto_1 | 54897.697 | -31.920 | 0.0001 | 2.089 |
|  |  |  |  |  |  |  |  | g__gut_metagenome | -1934.135 | -23.638 | 0.0001 | 1.662 |
|  |  |  |  |  |  |  |  | g__Lachnoclostridium | -9906.160 | -12.197 | 0.0001 | 2.379 |
|  |  |  |  |  |  |  |  | g__unidentified | -779.360 | -13.342 | 0.0001 | 1.538 |
|  |  |  |  |  |  |  |  | g__Ileibacterium | -2294.910 | -10.887 | 0.0001 | 1.803 |
|  |  |  |  |  |  |  |  | g__uncultured_rumen_bacterium | 13319.053 | 7.029 | 0.0001 | 1.606 |
|  |  |  |  |  |  |  |  | g__Odoribacter | 856.594 | 7.176 | 0.0001 | 1.540 |
|  |  |  |  |  |  |  |  | g__Helicobacter | -226.328 | -4.777 | 0.001 | 2.141 |
|  |  |  |  |  |  |  |  | g__Lachnospiraceae_UCG-001 | 272.926 | 2.878 | 0.018 | 1.665 |
| The dependent variable is represented by the abundance value of bacterial genera at the genus level:g_Odoribacter、g_Butyricimonas、g_Coprococcus、g_Oscillibacter、g_Desemzia、g_Bifidobacterium、g_Clostridium_sensu_stricto_1、g_Romboutsia、g_Ureaplasma、g_Eubacterium_siraeum_group、g_Acetitomaculum、g__Monoglobus、g__Psychrobacter、g__Rhodospirillum_sp._UNK.MGS-17、g__Ileibacterium、g__Tyzzerella、g__Coriobacteriaceae_UCG-002、g__Prevotellaceae_NK3B31_group、g_Lachnospiraceae_NK4B4_group、g_Adlercreutzia、g_unidentified、g_Bacteroides、g_Eubacterium_xylanophilum_group、g__Parasutterella、g__Lactococcus、g__Parabacteroides、g__Dubosiella、g__Citrobacter、g__Prevotella、g__Allobaculum、g__UBA1819、g__Streptococcus、g__ASF356、g__Lachnospiraceae_UCG-008、g__gut_metagenome、g__Corynebacterium、g__Rikenella、g__Rikenella_sp._Marseille-P3215、g__Parvibacter、g__Moryella、g__Escherichia-Shigella、g__Facklamia、g__Candidatus_Arthromitus、g__uncultured_Bacteroidales_bacterium、g__Marvinbryantia、g__Eubacterium_ruminantium_group、g__Desulfovibrio、g__Butyricicoccus、g_Lachnospiraceae_FCS020_group、g_A2、g__Lachnospiraceae_NC2004_group、g__Mycoplasma、g__Helicobacter、g__Lachnoclostridium、g__Harryflintia、g__Blautia、g__Akkermansia、g__Prevotellaceae_Ga6A1_group、g__UCG-005、g__Bilophila、g__Rodentibacter、g__Alistipes、g__Turicibacter、g__Ruminococcus、g__uncultured_rumen_bacterium、g__Aerococcus、g__Atopostipes、g__uncultured_Clostridia_bacterium、g__Anaeroplasma、g__Sporosarcina、g__Jeotgalicoccus、g__Christensenella_sp._Marseille-P2437、g__Paenalcaligenes、g__Acinetobacter、g__Roseburia、g__Caproiciproducens、g__Anaerovorax、g__Muribaculum、g__Prevotellaceae_UCG-001、g__Colidextribacter、g__Christensenellaceae_R-7_group、g__Gemella、g__Family_XIII_AD3011_group. | | | | | | | | | | | | |
