# Supplementary material for: Composition of gut microbiota involved in alleviation of dexamethasone-induced muscle atrophy by whey protein
Source: NPJ Sci Food. 2023 Nov 1;7:58. doi: 10.1038/s41538-023-00235-w (PMC10618183; doi:10.1038/s41538-023-00235-w)
Supplement: Supplementary file 2 — Reporting Summary [file 41538_2023_235_MOESM2_ESM.pdf]

Corresponding author(s): Guangxu Ren
Jiaqi Wang

Last updated by author(s): Oct 14, 2023

Reporting Summary

Nature Portfolio wishes to improve the reproducibility of the work that we publish. This form provides structure for consistency and transparency in reporting. For further information on Nature Portfolio policies, see our Editorial Policies and the Editorial Policy Checklist.

Statistics

For all statistical analyses, confirm that the following items are present in the figure legend, table legend, main text, or Methods section.

n/a	Confirmed	
☐	☒	The exact sample size (*n*) for each experimental group/condition, given as a discrete number and unit of measurement
☐	☒	A statement on whether measurements were taken from distinct samples or whether the same sample was measured repeatedly
☐	☒	The statistical test(s) used AND whether they are one- or two-sided<br>*Only common tests should be described solely by name; describe more complex techniques in the Methods section.*
☐	☒	A description of all covariates tested
☐	☒	A description of any assumptions or corrections, such as tests of normality and adjustment for multiple comparisons
☐	☒	A full description of the statistical parameters including central tendency (e.g. means) or other basic estimates (e.g. regression coefficient) AND variation (e.g. standard deviation) or associated estimates of uncertainty (e.g. confidence intervals)
☐	☒	For null hypothesis testing, the test statistic (e.g. *F*, *t*, *r*) with confidence intervals, effect sizes, degrees of freedom and *P* value noted<br>*Give P values as exact values whenever suitable.*
☒	☐	For Bayesian analysis, information on the choice of priors and Markov chain Monte Carlo settings
☒	☐	For hierarchical and complex designs, identification of the appropriate level for tests and full reporting of outcomes
☒	☐	Estimates of effect sizes (e.g. Cohen's *d*, Pearson's *r*), indicating how they were calculated

Our web collection on statistics for biologists contains articles on many of the points above.

Software and code

Policy information about availability of computer code

Data collection	no software was used
Data analysis	GraphPad Prism software version 8.0.0

For manuscripts utilizing custom algorithms or software that are central to the research but not yet described in published literature, software must be made available to editors and reviewers. We strongly encourage code deposition in a community repository (e.g. GitHub). See the Nature Portfolio guidelines for submitting code & software for further information.

Data

Policy information about availability of data

All manuscripts must include a data availability statement. This statement should provide the following information, where applicable:

- Accession codes, unique identifiers, or web links for publicly available datasets
- A description of any restrictions on data availability
- For clinical datasets or third party data, please ensure that the statement adheres to our policy

The sequence data have been deposited in the NCBI Sequence Read Archive under BioProject(PRJNA932250)

Research involving human participants, their data, or biological material

Policy information about studies with human participants or human data. See also policy information about sex, gender (identity/presentation), and sexual orientation and race, ethnicity and racism.

Reporting on sex and gender	Six-week-old male C57BL/6J Specific Pathogen Free (SPF) mice was used in this study.<br><br>The effects of long-term dexamethasone treatment were gender specific, with significantly fewer effects in females https://doi.org/10.1152/jappl.1997.82.1.125
Reporting on race, ethnicity, or other socially relevant groupings	NO available.
Population characteristics	NO available.
Recruitment	NO available.
Ethics oversight	NO available.

Note that full information on the approval of the study protocol must also be provided in the manuscript.

Field-specific reporting

Please select the one below that is the best fit for your research. If you are not sure, read the appropriate sections before making your selection.

- [x] Life sciences
- [ ] Behavioural & social sciences
- [ ] Ecological, evolutionary & environmental sciences

For a reference copy of the document with all sections, see nature.com/documents/nr-reporting-summary-flat.pdf

Life sciences study design

All studies must disclose on these points even when the disclosure is negative.

Sample size	The genetic background of mice was clear, and we used 10 mice in each group for the final experiment. According to the results of the preliminary experiment, there was no significant difference between the statistical power of 10 rats in each group and 20 rats in each group. Considering the operation and cost, 10 rats were selected to carry out the experiment.
Data exclusions	In all animals, we had no deaths or abnormalities throughout the experimental period, so all mice were included in the statistical analysis.
Replication	We set up ten animals in each group and repeated each experiment three times.
Randomization	All mice were randomly grouped after completion of adaptive feeding
Blinding	NO available.

Reporting for specific materials, systems and methods

We require information from authors about some types of materials, experimental systems and methods used in many studies. Here, indicate whether each material, system or method listed is relevant to your study. If you are not sure if a list item applies to your research, read the appropriate section before selecting a response.

Materials & experimental systems

n/a	Involved in the study	
☒	☐	Antibodies
☒	☐	Eukaryotic cell lines
☒	☐	Palaeontology and archaeology
☐	☒	Animals and other organisms
☒	☐	Clinical data
☒	☐	Dual use research of concern
☒	☐	Plants

Methods

n/a	Involved in the study	
☒	☐	ChIP-seq
☒	☐	Flow cytometry
☒	☐	MRI-based neuroimaging

Animals and other research organisms

Policy information about studies involving animals; ARRIVE guidelines recommended for reporting animal research, and Sex and Gender in Research

Laboratory animals	Six-week-old male C57BL/6J Specific Pathogen Free (SPF) mice
Wild animals	NO available.
Reporting on sex	male C57BL/6J mice
Field-collected samples	The mice were kept in a specific pathogen-free (SPF) mouse facility with a 12-hour light-dark cycle at a room temperature of 18-23? and 45% - 55% relative humidity for acclimatization.
Ethics oversight	All experimental procedures involving animals were performed in accordance with protocols approved by the Institutional Animal Care and Usage Committees (IACUC) of the Institute of Food and Nutrition Development, MARA. The approval number for these procedures is YYSLLSC20200047.

Note that full information on the approval of the study protocol must also be provided in the manuscript.
